# Examining characteristics of father-child relationship: A qualitative scoping review protocol

**DOI:** 10.12688/wellcomeopenres.23622.2

**Published:** 2026-01-22

**Authors:** SMITA TODKAR, Prashanth N Srinivas, Vikram Patel, Supriya Bhavnani

**Affiliations:** 1 Centre for Doctoral Studies, Manipal Academy of Higher Education, Manipal, Karnataka, 576104, India; 2 Child Development Group (CDG), Sangath, Porvorim, Goa, 403501, India; 3 Institute of Public Health Bengaluru, Bengaluru, Karnataka, 560070, India; 4 Department of Global Health and Social Medicine, Howard Hughes Medical Institute - Harvard Medical School, Boston, Massachusetts, USA

**Keywords:** Father, fathers/paternal involvement, child development, child mental health

## Abstract

**Background:**

Fathers’ participation in parenting directly contributes to child development and mental health. Emerging evidence demonstrates that high-quality paternal involvement leads to positive child social, emotional, psychosocial and developmental outcomes. Despite growing recognition of the importance of father-child relationships in child development and well-being, there remains limited understanding of the complex and multifaceted nature of this relationship, particularly in cultural, socioeconomic, and family structure contexts.

**Methods:**

This scoping review involves five stages: identifying research question/s, identifying relevant studies, study selection, data charting, collating, summarizing and reporting the results. An electronic database search will be performed on PubMed and PsycINFO using a combination of terms for the key phrases of father, involvement, child, child development and mental health in the title, abstract and keywords. Additionally, the ascendency approach will be used to look through other sources cited in the included studies. Rayyan software will be used to remove duplicates and complete the screening process. Data charting will be conducted using excel-spreadsheet. Two independent reviewers will screen 10% of records to achieve Inter-rater reliability (IRR) at each screening stage. Reviewers will conduct systematic data extraction independently using piloted forms, with discrepancies resolved through senior authors and team discussions along with the use of the Critical Appraisal Skills Programme (CASP) quality assessment tool.

**Results:**

The scoping review will identify characteristics of father-child relationships in early and middle childhood along with neurodiverse children. The reporting of findings will be structured according to the Preferred Reporting Items for Systematic reviews and Meta-Analysis extension for Scoping Reviews (PRISMA-ScR) checklist.

**Conclusions:**

The knowledge-base, synthesis by this review, will inform the development of a framework for paternal involvement that is applicable across majority family contexts. This framework has the potential to guide the formulation of contextually appropriate policies aimed at strengthening father's involvement and promoting child development and mental health, while identifying key gaps to inform future research.

## Plain language summary

The proposed scoping review in this protocol paper aims to explore how fathers’ involvement in parenting affects child development and mental health, particularly across different cultural, socioeconomic, and family contexts. Research has shown that when fathers are actively involved in their children's lives, it leads to positive outcomes in areas such as social skills, emotional well-being, and overall development. However, there is still limited understanding of the complex nature of father-child relationships, especially when considering diverse settings and family structures.

The proposed review will follow a PRISMA-ScR guidelines structured approach to gather and analyze studies on father-child relationships across early and middle age groups and settings, including rural and urban settings, as well as among neurodiverse children and aims to fill this gap in knowledge. The research will involve systematically searching of scientific databases for relevant studies, focusing on keywords related to fathers, involvement, child development, and child mental health. A team of independent reviewers will screen and select studies based on specific inclusion and exclusion criteria to ensure the quality of the review.

The goal of this review is to identify the key characteristics of father-child relationships during early and middle childhood, while also considering how paternal involvement differs in high versus low-resource settings and among neurotypical and neurodiverse children. The findings will be summarized according to established guidelines, and a clear (PRISMA-ScR) flow diagram will outline the process used to include and exclude studies.

Ultimately, the review aims to create a better understanding of how fathers’ involvement varies across different contexts, which can inform policies and practices to support fathers in their parenting roles. The review will also highlight areas that require further research, ultimately helping to improve child development and mental health.

## Introduction

Early experiences in households and communities influence the developing brain, supporting the development of a range of early skills such as cognitive, social, executive function and self-regulation skills ( Gibson, 2014; Murray-Kolb *et al.*, 2014; Rollè *et al.*, 2019). The Nurturing Care Framework provides impetus to parents and caregivers, researchers, academics, governments and civil society groups to ensure care for every infant and young child such that they can get the best start in life ( World Health Organization, 2018a). The framework consists of five components: adequate nutrition, responsive caregiving, security and safety, opportunities for early learning and good health ( World Health Organization, 2018b). Majority of the evidence generated about the impact of parenting on early childhood focuses on maternal involvement but paternal (fathers’) involvement is also an important aspect of nurturing care ( Inbaraj *et al.*, 2020; Mithra *et al.*, 2021).

Fathers’ participation in parenting directly contributes to children’s development and mental health ( Rollè *et al.*, 2019). Emerging literature demonstrates that positive child social, emotional, psychosocial and developmental outcomes are associated with high-quality paternal involvement ( Abraham *et al.*, 2021; Diniz *et al.*, 2021; Gibson, 2014; Kenigsberg *et al.*, 2016; Solichah *et al.*, 2025). However, even though paternal involvement is important for child development, fathers face numerous barriers to engaging with children such as cultural norms, maternal gatekeeping and financial responsibilities ( Maselko *et al.*, 2019; Nair *et al.*, 2020). These barriers are contextual, and need local solutions to enhance paternal involvement and thereby positively impact children’s well-being.

Paternal involvement has been characterised into frameworks since the late 1980’s. The initial framework by Lamb and colleagues ( Lamb *et al.*, 1985) emphasized paternal involvement in a) interactions: the father’s role of managing the family and their involvement in family rituals such as having at least one meal together or participating in festivals, b) availability: the extent to which fathers are accessible to their children, and c) responsibility: defined as ‘the extent of which fathers take responsibility for the care and well-being of their children’. A few years later Parke's *et al.* ( Parke, 1995) published a framework with five categories; which are play, caregiving, guidance, supporting socialization with peers, and influencing their cognitive development. In early 21 ^st^ century, the conceptualisation of paternal involvement evolved as Hawkins’ *et al.*, 2002 published a seven global indicators of fathers involvement and therefore framework comprising provision refers to father’s role in providing child’s physical and economic needs, protection refers to safeguarding the child’s physical and emotional well-being, companionship encompasses father’s role in spending time with the child, tutelage involves father’s role in teaching and guiding the child, role-modelling for fathers serve as role models and influence child’s behaviour and values, turning in refers to father’s emotional connect with their child for understanding and responding to their needs and lastly mentorship refers to father’s role as mentor and guide for the child ( Hawkins *et al.*, 2002). In the 2010, Pleck's framework re-conceptualized paternal involvement by clarifying the quality of father child interactions such as positive engagement activities, warmth and responsiveness and control, along with two auxiliary components of indirect care and process responsibility ( Pleck, 2010; Pleck, 2012).

Harrington and colleagues ( Harrington, 2022) highlight the role of fathers in the 21st century as being a combination of their responsibilities at the workplace and simultaneously being caregivers at home for their children. They define the term ‘good father’ as one who includes caregiving characteristics such as providing love and emotional support, being a teacher and guide and contributing to daily tasks of childcare ( Harrington, 2022). The COVID-19 pandemic further emphasized the role of fathers and the importance of both parents and family dynamics as important considerations for providing ‘Nurturing care’ to children were highlighted ( Dol *et al.*, 2024; Dungan *et al.*, 2023). The evolution of these frameworks over the last 50 years underscores the changing narratives of fathers’ involvement.

Recently, there has been an effort to synthesize the evidence-base on numerous aspects of paternal involvement. Lut *et al.*, 2022 conducted a systematic scoping review of studies of fatherhood that used linked administrative data to their children and studies outcomes were related to child health and development ( Lut *et al.*, 2022). This review identifies methods that has been used globally to link fathers and children in administrative data and unable to identify direct measures of paternal involvement. This review also created mapping linkage methods to the paternal involvement framework with possible proxies. Diniz *et al.*, 2021 conducted a systematic review of qualitative studies examining father involvement during early childhood. This review highlighted the significance of paternal engagement in shaping child development outcomes, revealing themes such as emotional support, playfulness, and co-parenting dynamics ( Diniz *et al.*, 2021). The authors emphasized the importance of contextual factors, including cultural background and family structure, in influencing father involvement. Both the reviews emphasize the importance of accurate and comprehensive data on father-child relationships for informed policy and practice decisions along with the need for further research on the complexities of father-child relationships, particularly in diverse cultural and socioeconomic contexts ( Diniz *et al.*, 2021; Lut *et al.*, 2022).

Despite the growing recognition of the importance of father-child relationships in child development and well-being, there remains limited understanding of the complex and multifaceted nature of this relationship, particularly in diverse cultural, socioeconomic, and family structure contexts. Existing literature reviews have primarily focused on specific aspects of father involvement, such as paternal engagement or attachment and that too during early childhood, but a comprehensive synthesis of the empirical evidence on the characteristics of father-child relationships across various contexts (age of child, various settings like rural, urban, tribal etc.) is lacking. Specifically, there is a need to identify and categorize the key dimensions of father-child relationships along with the influence of sociodemographic factors (e.g., culture, socioeconomic status, family structure). It is also important to investigate the influence of father-child relationships on child outcomes in early and middle childhood (e.g., age appropriate cognitive, emotional, behavioural development). This scoping review aims to address this knowledge gap by qualitatively mapping the empirical qualitative and mixed methods research studies on the characteristics of father-child relationships in early and middle childhood, including research that considers how neurodiverse children’s developmental trajectories may differentially shape father-child interactions. The inclusion of this subgroup is intended to determine whether and how the existing literature addresses differentiated needs. Consistent with the purpose of scoping reviews, the focus is on capturing the breadth of existing evidences rather than conducting in-depth comparative analysis. The findings will provide a foundation for future research and inform the development of potential evidence-based interventions aimed at strengthening paternal involvement and promoting optimal child development and mental health.

## Ethics and dissemination

This scoping review involves synthesis of existing literature and hence the ethics approval is not required. The dissemination plan is to publish the findings in peer-reviewed journal and present it in relevant conferences to inform researchers, students, policy makers and healthcare professions especially working in the field of parental involvement and child development.

## Methods

### Objectives

The objective of this scoping review is to examine the characteristics of father-child relationship which influence child development and mental health and to determine barriers and facilitators of paternal involvement.

### Eligibility criteria

Study characteristics. The Population, Concept and Context (PCC) framework will be used to guide the development of this scoping review literature search strategy. The Population (P) for this review is fathers of children aged 0 to 12 years. The 0–12 years of age range is selected based on established developmental frameworks describing early childhood (0–5), middle childhood (6–9), and late childhood (10–12) before the onset of adolescence ( Berkel *et al.*, 2018; Santrock, 2019) The World Health Organization (WHO) categorises adolescence as beginning at 10 years, but also acknowledges that the ages 10–12 represent a transitional period from late childhood into early adolescence, with substantial overlap for growing autonomy but continued depence on parental support ( *Guidelines on Mental Health Promotive and Preventive Interventions for Adolescents*, 2020). The Concept (C) for this review is father-child relationship which includes various aspects such as availability of father, interaction, various forms of engagement (direct, indirect, positive, negative), influence on the child and their sense of responsibility. The Contextual (C) factors for this review are research articles published in last 20 years (2004–2024), articles published using qualitative and mixed-methods as research methods in any settings (rural, urban, high-income, low-income), articles published having abstract available in English language and articles in any language.

### Study design

The scoping review will be conducted with the following stages which are described below:

1.   Identifying research question/s

2.   Identifying relevant studies

3.   Study selection

4.   Charting the data

5.   Collating, summarising and reporting the results

( Arksey & O’Malley, 2005). The publication of the scoping review will report if there were any modifications or deviations from the published protocol.

***Stage 1: identifying the research questions***

The scoping review primary question is, How does existing literature of paternal involvement characterize father – child relationships?

Secondary questions for this scoping review are as follows:

1.   What are the socio-demographic determinants of paternal involvement?

2.   How does paternal involvement differ in high resource settings compared to low resource settings?

3.   How does paternal involvement differ in early compared to middle childhood?

4.   How does paternal involvement differ among neurodiverse children?

***Stage 2: Identifying relevant studies***

All the publications retrieval will occur from databases PubMed and PsycINFO. In addition to PubMed and PsycINFO, we included Scopus as a third database. When we did the search on PubMed and PsycINFO we had total 9985 articles and Scopus gave us 9427. However, we found that all articles retrieved from Scopus were duplicates of those already identified in PubMed and PsycINFO. As balance between feasibility and comprehensiveness, we therefore restricted our search to these two databases. The review will include all the publications from the 01-01-2004 till 01-04-2024 (~20 years). Additionally, ascendency approach will be used to look through other sources cited in the included studies. The ~ 20-year window (2004-2024) was deliberately selected based on the significant conceptual and sociocultural shifts in father involvement since the early 2000s ( Lamb, 2010; Moore, 2003). Research emerging from this period reflects more nuanced and comprehensive understandings of fatherhood, including increased paternal engagement, the development of co-parenting frameworks, and evolving gender norms ( Bronstein, 2006; Bronte-Tinkew *et al.*, 2009; Lamb, 2010; Moore, 2003). Conference abstracts, reviews, theses, dissertations and project reports will not be included in this scoping review.

***Stage 3: Study selection***

In this review duplicate records will be removed to ensure that we assess all the studies only once. We will first screen titles, then abstracts and finally full texts of identified studies using Rayyan ( Ouzzani, 2016), a systematic review management software. In screening process, we will rigorously apply inclusion and exclusion criteria.

***Stage 4: Charting the data***

A standardized data extraction form will be developed to systematically extract the required data from the included studies. This form will cover the following aspects of the research papers:

1.   Metadata

a.   study design;

b.   geographical location of study and settings e.g. urban or rural or tribal;

c.   years in which study was conducted;

d.   study sponsorship;

2.   Study details

a.   study objectives;

b.   recruitment strategy

c.   sample size, including attrition rates and reasons;

3.   Participants demographic – which may include age of the child, socioeconomic status, education level of child and parents, neurodiversity among children, and ethnicity;

4.   Qualitative data fragments

a.   Themes of father-child involvement, which may include the availability of father, time spent with the child, types of engagement (direct, indirect, positive, negative), any control practices and sense of responsibility among father ( Hawkins *et al.*, 2002; Lamb *et al.*, 1985; Parke, 2000; Pleck, 2010)

b.   Inductive approach will be followed and themes relevant to scoping review research question/s will be added.

***Stage 5: Collating, summarising and reporting the results***

Existing characteristics and their definitions of father-child relationship will be identified from included papers, the definitions and emerged themes will be collated and mapped against each other (outlined above in stage 4). Analytical data of interest will include the details of authorship and year of publication to identify trends of father-child relationship throughout the years. The outlined domains of the characteristics of father-child relationship will be summarised in the table format. Data will be presented in summary format in order to address the exploratory nature of the research questions. The reporting of findings will be structured according to the Preferred Reporting Items for Systematic reviews and Meta-Analysis extension for Scoping Reviews (PRISMA-ScR) checklist (PRISMA; Moher *et al.*, 2015). A PRISMA flow diagram will be included in the final report which will outline the process of identifying, screening, including and excluding studies.

**Study setting.** The review will include primary research studies conducted in any settings (high-income, low-income, urban-rural) with abstract published in English language

### Inclusion and exclusion criteria

The inclusion criteria will be

a.   Studies that examine paternal involvement

b.   Studies published in last 20 years (01-01-2004 to 01-04-2024)

c.   Studies in which child-age is between 0 to 12 years (early to late childhood)

d.   Studies using only qualitative or mixed methods

The exclusion criteria will be

a.   Studies where only maternal involvement is examined

b.   Studies in families with multiple fathers

c.   Studies examining fathers’ involvement only during adolescence (>12-year-olds), or in children beyond the desired age-range and not providing details disaggregated by age

d.   Studies which only examine fathers’ involvement during labour and hospitalization period or any physical disease condition of the child

e.   Studies describing implications of fathers’ involvement only on child physical growth

f.   Studies describing involvement of fathers experiencing any physical or mental health condition

g.   Case studies – sample size less than 5 ( Isman *et al.*, 2013)

### Key outcomes

This scoping review will report the characteristics of father-child relationship, across the entire range of childhood and amongst neurotypical and neurodiverse children. It will build a framework based on the thematic analysis of extracted data and examine the facilitators and barriers for paternal involvement.

### Search strategy

The search strategy was designed by applying PPC framework for scoping review which included relevant terms for each key phrase of father, child, involvement, child development and mental health with Boolean operators (See Table 1). Following this, the search strategy was tailored to suit the specific requirements of each database included in this review. The iterative process involved adjusting search syntax and refining search strategy to optimize retrieval of relevant studies. Details of the search on each database will be documented to include information on the number of hits, retrievable studies and keywords used. Table 2 presents the search strategy used in PubMed and PsycINFO.

**Table 1.** Relevant key phrases and search terms.

Sr. No.	Key phrase	Search terms
1	Father	father* OR paternal OR dad
2	Involvement	involve* OR engage* OR availab* OR responsib* OR play* OR warm* OR care* OR contact* OR support* OR communicat* OR teach* OR emoti*
3	Child	child*
4	Child development and mental health	develop* OR movement OR progress* OR cogniti* OR social* OR motor OR language* OR adapt* OR mental health OR psycho* OR behav* OR adjust* OR attention
5	Limits	Child (0–12 years) Human Year= ‘1 January 2004 to 1 April 2024 (current)’

**Table 2.** Search strategy in used PubMed and PsycINFO database.

PubMed	PsycINFO
(father*[Title/Abstract] OR paternal[Title/Abstract] OR dad[Title/Abstract]) AND (involve*[Title/Abstract] OR engage*[Title/Abstract] OR availab*[Title/Abstract] OR responsib*[Title/Abstract] OR play*[Title/Abstract] OR warm*[Title/Abstract] OR care[Title/Abstract] OR contact*[Title/Abstract] OR support*[Title/Abstract] OR communicat*[Title/Abstract] OR emoti*[Title/Abstract]) AND (child*[Title/Abstract]) AND (develop*[Title/Abstract] OR movement [Title/Abstract] OR progress*[Title/Abstract] OR cogniti*[Title/Abstract] OR social*[Title/Abstract] OR motor[Title/Abstract] OR language*[Title/Abstract] OR adapt*[Title/Abstract] OR mental health[Title/Abstract] OR psycho*[Title/Abstract] OR behav*[Title/Abstract] OR adjust*[Title/Abstract] OR attention[Title/Abstract])	(Title: father* OR Title: paternal OR Title: dad) AND (Abstract: father* OR Abstract: paternal OR Abstract: dad) AND (Title: involve* OR Title: engage* OR Title: availab* OR Title: responsib* OR Title: play* OR Title: warm* OR Title: care OR Title: contact* OR Title: support* OR Title: communicat* OR Title: emoti*) AND (Abstract: involve* OR Abstract: engage* OR Abstract: availab* OR Abstract: responsib* OR Abstract: play* OR Abstract: warm* OR Abstract: care OR Abstract: contact* OR Abstract: support* OR Abstract: communicat* OR Abstract: emoti*) AND (Title: child*) AND (Abstract: child*) AND (Title: develop* OR Title: movement OR Title: progress* OR Title: cogniti* OR Title: social* OR Title: motor OR Title: language* OR Title: adapt* OR Title: mentalhealth OR Title: psycho* OR Title: behav* OR Title: adjust* OR Title: attention) (Abstract: develop* OR Abstract: movement OR Abstract: progress* OR Abstract: cogniti* OR Abstract: social* OR Abstract: motor OR Abstract: language* OR Abstract: adapt* OR Abstract: mentalhealth OR Abstract: psycho* OR Abstract: behav* OR Abstract: adjust* OR Abstract: attention) AND Age Group: Infancy (0–23 months) OR Preschool Age (2–5 years) OR School Age (6–12 years) AND Year: 2004 To 2024

### Data management

For removing duplicates, screening and reviewing Rayyan ( Ouzzani, 2016), a systematic review management software will be used. Data charting will be done using Microsoft Excel ( Microsoft Corporation, 2019). Additionally, Critical Appraisal Skills Programme (CASP) quality checklists will be used as quality assessment tool for the research articles ( CASP(Qualitative studies) Checklist, 2023)

### Screening and data extraction

Screening will be done by two independent reviewers (ST, NM) and Inter-Rater Reliability (IRR) will be established at each screening (title, abstract and full text) stage. A minimum of 10% records will be screened together to align on inclusion/exclusion criteria and then 10% articles will be rated by both reviewers while remaining blind to each other’s decisions at every round of screening. Agreement will be calculated using the Cohen’s Kappa coefficient (K) statistics ( McHugh, 2012) using ‘R software’ ( R Core Team, 2024). We expected to achieve minimum substantial agreement (i.e. 0.6 and above). If in case the K is not achieved then we will repeat the IRR on next 10% randomly assigned articles. The remaining records will be equally divided between the two reviewers. At each stage, all discrepancies among the reviewers will be discussed and resolved by senior authors (SB, PNS). The reasons for exclusion will be recorded at the full-text screen stage and reported. The screening process will be presented using PRISMA flow diagram.

In the first stage of data extraction, a standardized data extraction form will be developed to systematically and manually extract the required data from the included studies. The form will be initially piloted on 10 included studies by two reviewers (ST, NM) and iteratively improved to ensure its accuracy and comprehensiveness. Included studies will be divided equally for data to be exacted by both reviewers independently. Each reviewer’s extracted data will be reviewed by the other reviewer after completion and disagreements will be resolved through mutual discussion and with the guidance from senior expert author/s (SB, PNS). Throughout the data extraction process reviewers will ensure all the relevant data shall be captured, and all authors are aware of the progress.

In the second stage of the data extraction the CASP quality assessment tool will be used which will assess the quality of included sources ( CASP(Qualitative studies) Checklist, 2023). All the studies will be rated together by two reviewers (ST, NM) with detailed discussion and discrepancies will be resolved through consultation with senior expert author/s (SB, PNS). All eligible studies will be included in the review, including those assessed as having a high risk of bias using the CASP tool, as their findings may still offer valuable insights into the characteristics, contexts, and conceptualisations of father involvement within specific geographical and cultural settings. To ensure transparency and support appropriate interpretation of the findings, risk-of-bias assessments will be reported alongside each study, and any limitations identified through CASP will be clearly highlighted.

### Data synthesis and analysis

Themes and patterns will be synthesised from the extracted data on characteristics of father-child relationship for the primary research question. Initially findings from each study will be coded to identify concepts relevant to the father-child relationships. These codes will then be grouped into descriptive themes that reflect recurring patterns across studies, which might include bonding, preferred activities for spending time together, handling behavioural challenges and being the provider for the child. The presentation of data will be done using descriptive methods which will include figures and tables for clarity of representation. In the next step reviewers will examine these studies to explore how socio-demographic factors are described, experienced and understood within father-child relationships, as well as how paternal involvement differs across high vs low-resource settings, early vs late childhood and among neurotypical vs neurodiverse children, in order to address the secondary research questions of this review.

The Preferred Reporting Items for Systematic Reviews and Meta-Analyses (PRISMA-ScR) guidelines extension for scoping reviews ( Moher *et al.*, 2015; Tricco *et al.*, 2018) will be followed to summarize the identified characteristics of father-child relationship. The key themes and types of paternal involvement observed across included studies will be highlighted. In the next steps a framework for rural households will be designed based on the emerged themes of father-child relationship from this scoping review.

### Study status

This scoping review is currently in the third stage, study selection based on inclusion and exclusion criteria.

### Protocol and registration

The final protocol is registered with the Open Science Framework in October 2024; it is accessible with the DOI https://doi.org/10.17605/OSF.IO/CWBG6

## Conclusion

The review will contribute to the understanding of the characteristics of father-child relationship across early, middle and late childhood and it will differentiate paternal involvement in high vs low-resource settings and among neurotypical vs neurodiverse children. The knowledge-base, synthesised by this review, will inform the development of a framework for paternal involvement that is applicable across majority family context. This framework has the potential to guide the formulation of contextually appropriate policies aimed at strengthening father’s involvement and enhancing child development and mental health, while identifying key gaps to inform future research. This framework will be applied to examine paternal involvement in the established ‘Sustainable Programme Incorporating Nutrition and Games (SPRING) cohort’ at rural households of Rewari district of Haryana, India ( Bhopal *et al.*, 2018; Kirkwood *et al.*, 2023) through the ongoing COINCIDE study ( Lobo *et al.*, 2024).

## Ethics and consent

Ethical approval and consent were not required.

## Data Availability

No underlying data is associated with this article. OSF Registries: Characteristics of father-child relationship: A scoping review protocol https://doi.org/10.17605/OSF.IO/CWBG6 ( Todkar *et al.*, 2024) This project contains the following extended data: Search strategy.pdf Data are available under the terms of the Creative Commons Zero “No rights reserved” data waiver (CC0 1.0 Public domain dedication)

## Actual start date

01 March 2024

## Anticipated completion date

28 February 2025
