# Supplementary material for: Examining characteristics of father-child relationship: A qualitative scoping review protocol
Source: Wellcome Open Res. 2026 Jan 22;10:124. Originally published 2025 Mar 5. [Version 2] doi: 10.12688/wellcomeopenres.23622.2 (PMC12921393; doi:10.12688/wellcomeopenres.23622.2)
Supplement: Peer review history

# Reviewer report: Reviewer response for version 2

**DOI:** 10.21956/wellcomeopenres.28453.r145528

**Published:** 2026-02-19

**Authors:** Kristin Hadfield

**Affiliations:** 1 Trinity College Dublin, Dublin, Ireland

The authors have adequately addressed my concerns about the review protocol.

Is the study design appropriate for the research question?

Partly

Is the rationale for, and objectives of, the study clearly described?

Partly

Are sufficient details of the methods provided to allow replication by others?

Partly

Are the datasets clearly presented in a useable and accessible format?

Not applicable

Reviewer Expertise:

child and adolescent mental health, wellbeing, family relationships, global mental health, resilience

I confirm that I have read this submission and believe that I have an appropriate level of expertise to confirm that it is of an acceptable scientific standard.

# Reviewer report: Reviewer response for version 1

**DOI:** 10.21956/wellcomeopenres.26062.r137647

**Published:** 2025-11-26

**Authors:** Kristin Hadfield

**Affiliations:** 1 Trinity College Dublin, Dublin, Ireland

This is a protocol for a potentially interesting review on characteristics of father involvement which influence children's development and mental health (including wellbeing), as well as the barriers and facilitators of such involvement. However, I think there are a number of critical issues with the proposed scoping review which will limit the utility of the review.

 I don't think that the search strategy is sufficiently strong. There is only a plan to search two databses, and then only studies with the search terms in the title/abstract/keywords. It is only from the past 20 years, but no rationale is provided for this restriction. Similarly, it is focused on children aged 0-12 years, but no explanation is provided for why this age range (it's said that it goes to "late childhood", but what framework are you basing 12 years old as "late childhood" from, for instance?). There is no engagement with the grey literature despite there being NGO reports etc on this topic. I would expect that a scoping review would have a more robust search strategy, with more databases, inclusion of grey literature, explanation of key decisions around time and age restrictions, etc. 

 I am also concerned that the focus of the review is quite broad. "Characteristics of father-child relationships" is non-specific, and the decision to focus on "diverse settings" (e.g. all settings) and then all children but with one question focused on neurodiverse children is not well-justified. The focus on all characteristics of father-child relationships in all countries is extremely wide and I am concerned that the resulting scoping review will not be able to be sufficiently focused to provide a clear output. 

 I don't think that "diverse backgrounds" is really the correct phrasing for the fathers/children included in this study. It's ultimately going to include any research conducted in any setting, and so it's not targeting some kind of diverse focus. They may be diverse or not, depending on if you only get studies from certain locations or types of fathers included. 

 Is a review incorporating only qualitative and mixed methods projects really suitable to answer the question: "What are the socio-demographic determinants of paternal involvement?"

 If a study is considered to have a high risk of bias from CASP, will it be included in the scoping review or no? Some explanation of this is needed.

 Small points:

 - Appears to be a typo here: "This scoping reviewinvolves five stages"

 - I think "The" or "An" is missing here: "Electronic database search will be performed on PubMed and PsycINFO..."

 - Research from more than 10 years ago is not really "emerging literature": "Emerging literature demonstrates that positive child social, emotional, psychosocial and developmental outcomes are associated with high-quality paternal involvement ( Gibson, 2014)." also, there is a long-standing and rapidly growing body of work on this topic which this sentence ignores. 

 - "the conceptualisation of paternal involvement evolved as Hawkins’ *et al.* ( Hawkins *et al.*, 2002)" not correct formatting for the reference. Similar issue here: "Lut *et al.* ( Lut *et al.*, 2022)" and "Diniz E *et al.* ( Diniz *et al.*, 2021)" etc

 - Typo here: "such as paternal engagement or attachment and that too during early-childhood"

 - Typo here: "Participants demographics"

Is the study design appropriate for the research question?

Partly

Is the rationale for, and objectives of, the study clearly described?

Partly

Are sufficient details of the methods provided to allow replication by others?

Partly

Are the datasets clearly presented in a useable and accessible format?

Not applicable

Reviewer Expertise:

Adolescent development, family processes, resilience, displaced youth, mental health, environmental influences

I confirm that I have read this submission and believe that I have an appropriate level of expertise to state that I do not consider it to be of an acceptable scientific standard, for reasons outlined above.

# Response

**Published:** 2026-01-11

**Authors:** SMITA TODKAR

**Affiliations:** Child Development Group (CDG), Sangath, Porvorim, Goa, India

**Response to Reviewer 2 – Scoping Review Protocol Paper**

 This is a protocol for a potentially interesting review on characteristics of father involvement which influence children's development and mental health (including wellbeing), as well as the barriers and facilitators of such involvement. However, I think there are a number of critical issues with the proposed scoping review which will limit the utility of the review. Thank you very much for your thoughtful and constructive feedback on our protocol. We appreciate your careful review and the opportunity to clarify and strengthen several aspects of our methodological approach for this scoping review. Please find our response to your comments below:

 1. I don't think that the search strategy is sufficiently strong. There is only a plan to search two databases, and then only studies with the search terms in the title/abstract/keywords. Response: Our initial decision to focus on three core databases was guided by their relevance to developmental psychology, family studies, and child mental health. In addition to PubMed and PsycINFO, we included Scopus as a third database. When we did the search on PubMed and PsycINFO we had total 9985 articles and Scopus gave us 9427. However, we found that all articles retrieved from Scopus were duplicates of those already identified in PubMed and PsycINFO. Methodological guidance indicates that scoping reviews may tailor the number of databases based on topic saturation, feasibility, and preliminary mapping (Arksey & O’Malley, 2005; Levac et al., 2010; S. S. Peters, 2021). Consistent with this, our preliminary scoping searches suggested that PubMed and PsycINFO captured a substantial proportion of relevant studies on father involvement. The use of title, abstract, and keyword searches aligns with established scoping review methodology, as it balances search sensitivity with relevance  (Arksey & O’Malley, 2005; Moher et al., 2015; M. D. J. Peters et al., 2020; Tricco et al., 2018). The PRISMA-ScR reporting guidelines support this approach, including the use of synonyms and Boolean operators such as “AND” and “OR” (Tricco et al., 2018). Accordingly, these methods are being applied in the present review and will be transparently reported in the final scoping review manuscript. The changes made for 1 in the manuscript were as follows (Page 9-10, line 210-215): In addition to PubMed and PsycINFO, we included Scopus as a third database. When we did the search on PubMed and PsycINFO we had total 9985 articles and Scopus gave us 9427. However, we found that all articles retrieved from Scopus were duplicates of those already identified in PubMed and PsycINFO. As balance between feasibility and comprehensiveness, we therefore restricted our search to these two databases.

 2. It is only from the past 20 years, but no rationale is provided for this restriction. Response: In a book documenting the historical evaluation of fatherhood, Lamb notes significant conceptual and sociocultural shifts in fathers' involvement over time (Lamb, n.d.-a). Research emerging from the last 20 years reflects more nuanced and comprehensive understandings of fatherhood, including increasing paternal engagement, the development of co-parenting frameworks, and evolving gender norms (Bronstein, 2006; Bronte-Tinkew et al., 2009; Lamb, n.d.-a). We have incorporated this rationale into the manuscript. The changes made for 1 and 2 in the manuscript were as follows (Page 9-10, line 217-223): The review will include all the publications from the 01-01-2004 till 01-04-2024 (20 years). The 20-years window (2004-2024) was deliberately selected based on the significant conceptual and sociocultural shifts in father involvement since the early 2000s (Lamb, n.d.-a; Moore, 2003). Research emerging from this period reflects more nuanced and comprehensive understandings of fatherhood, including increased paternal engagement, the development of co-parenting frameworks, and evolving gender norms (Bronstein, 2006; Bronte-Tinkew et al., 2009; Lamb, n.d.-a; Moore, 2003). Additionally, ascendency approach will be used to look through other sources cited in the included studies. Conference abstracts, reviews, theses, dissertations and project reports will not be included in this scoping review.  

 3. Similarly, it is focused on children aged 0-12 years, but no explanation is provided for why this age range (it's said that it goes to "late childhood", but what framework are you basing 12 years old as "late childhood" from, for instance?). Response: The 0-12 years age range was selected based on established developmental frameworks describing early childhood (0-5), middle childhood (6-9) and late childhood (10-12) before the onset of adolescence (Berkel C et al., 2018; Santrock, 2019). The World Health Organization (WHO) categorises adolescence as beginning at 10 years, but also acknowledges that the ages 10–12 represent a transitional period from late childhood into early adolescence, with substantial overlap for growing autonomy but continued dependence on parental support (Guidelines on Mental Health Promotive and Preventive Interventions for Adolescents, 2020). Our intention was to focus on developmental stages where parental involvement—particularly father involvement—has direct and foundational influence on socio-emotional and mental health outcomes. We have added this in the protocol to explicitly reference these developmental definitions. The changes made for 3 in the manuscript were as follows (Page 8, line 176-183): Study characteristics. The Population, Concept and Context (PCC) framework will be used to guide the development of this scoping review literature search strategy. The Population (P) for this review is fathers of children aged 0 to 12 years. The 0–12 age range is selected based on established developmental frameworks describing early childhood (0–5), middle childhood (6–9), and late childhood (10–12) before the onset of adolescence (Berkel C et al., 2018; Santrock, 2019). The World Health Organization (WHO) categorises adolescence as beginning at 10 years, but also acknowledges that the ages 10–12 represent a transitional period from late childhood into early adolescence, with substantial overlap for growing autonomy but continued dependence on parental support ( *Guidelines on Mental Health Promotive and Preventive Interventions for Adolescents*, 2020).

 4. There is no engagement with the grey literature despite there being NGO reports etc on this topic.  Response: The decision not to include grey literature was based on two considerations. First, established scoping review guidelines recommend the inclusion of grey literature primarily when it is relevant and when peer-reviewed evidence is limited (Arksey & O’Malley, 2005; Levac et al., 2010; M. D. J. Peters et al., 2015, 2020; Tricco et al., 2018). In the present review, a substantial body of peer-reviewed literature was identified, with over 9,000 articles addressing fathers’ involvement. Second, the grey literature examined largely drew upon peer-reviewed studies that were already captured in the review. Therefore, to avoid unnecessary duplication and given the comprehensive coverage of peer-reviewed sources, grey literature was not included in this scoping review. 

 5. I am also concerned that the focus of the review is quite broad. "Characteristics of father-child relationships" is non-specific, and the decision to focus on "diverse settings" (e.g. all settings) and then all children but with one question focused on neurodiverse children is not well-justified. The focus on all characteristics of father-child relationships in all countries is extremely wide and I am concerned that the resulting scoping review will not be able to be sufficiently focused to provide a clear output.  Response: Thank you for raising this point regarding the breadth of the review. While we recognise that “characteristics of father–child relationships” is inherently broad, this breadth is intentional and aligns with the purpose of a scoping review—to map the range, nature, and conceptualisations of a topic that is currently very different across disciplines, populations, and settings. Existing literature shows that father involvement and father–child relationships differ across cultural, socioeconomic, and policy environments (Lamb, n.d.-b; Moore, 2003). Given this variation, narrowing the scope too early will risk excluding relevant constructs or frameworks that have been operationalised differently across global contexts. Our inclusion of ‘diverse settings’ trying to reflect the recognition that father involvement is shaped by contextual and structural factors. The goal is not to generate cross-cultural comparisons, but to map what is known across contexts and identify gaps that may be setting-specific. We made this clearer in the revised protocol by specifying that our aim is ‘breadth of mapping’, not depth of comparative analysis.  Regarding neurodiverse children, our intension was to ensure that the review remains inclusive of populations whose developmental trajectories may influence father-child interactions differently and thus it is included as a secondary research question. In the revised protocol, we emphasised that this subgroup is included to determine whether the literature addresses differentiated needs, rather than as a separate primary focus.  Although the scope of this review is broad, the questions are structured to allow the mapping of evidence into organised domains (e.g., father-child characteristics, change in fathers’ involvement over the years of child age). Scoping review methodology explicitly supports broad initial scopes when the field is conceptually diffuse, with the goal of generating a structured overview to guide future, more focused systematic reviews (Arksey & O’Malley, 2005; Munn et al., 2018). The changes made for 5 in the manuscript were as follows:

1. This scoping review aims to address this knowledge gap by qualitatively mapping the empirical qualitative and mixed methods research studies that examine the characteristics of father-child relationships in early and middle childhood, including research that considers how neurodiverse children’s developmental trajectories may differentially shape father–child interactions. The inclusion of this subgroup is intended to determine whether and how the existing literature addresses differentiated needs. Consistent with the purpose of scoping reviews, the focus is on capturing the breadth of existing evidences rather than conducting an in-depth comparative analysis. The findings will provide a foundation for future research and inform the development of potential evidence-based interventions aimed at strengthening paternal involvement and promoting optimal child development and mental health. (Page 7, line 154-161)
2. Themes and patterns will be synthesised from the extracted data on characteristics of father-child relationship for the primary research question. Initially findings from each study will be coded to identify concepts relevant to the father-child relationships. These codes will then be grouped into descriptive themes that reflect recurring patterns across studies, which might include bonding, preferred activities for spending time together, handling behavioural challenges and being the provider for the child. (Page 18, line 340-345)

6. I don't think that "diverse backgrounds" is really the correct phrasing for the fathers/children included in this study. It's ultimately going to include any research conducted in any setting, and so it's not targeting some kind of diverse focus. They may be diverse or not, depending on if you only get studies from certain locations or types of fathers included.  Response: Thank you for this helpful observation. We agree that the term “diverse backgrounds” may imply a targeted focus that is not fully aligned with our broader inclusion strategy. Our intention was not to suggest that the review specifically seeks out studies of fathers or children from under-represented or socio-culturally diverse groups. Rather, the review aims to capture evidence across the full range of contexts in which research on father–child relationships has been conducted. These contexts may vary widely—or may not be diverse at all—but all eligible studies will be included to reflect the breadth of geographical, cultural, and social environments in which father–child relationships have been examined. We acknowledge your comment and following are the changes made for 6 in the manuscript:

1. Examining characteristics of father-child relationship: A qualitative scoping review protocol (Page 1, line 1-2)
2. The scoping review will identify characteristics of father-child relationships in early and middle childhood along with neurodiverse children. (Page 2, line 37)
3. The knowledge-base, synthesised by this review, will inform the development of a framework for paternal involvement that is applicable across majority family contexts. This framework has the potential to guide the formulation of contextually appropriate policies aimed at strengthening father’s involvement and promoting child development and mental health, while identifying key gaps to inform future research. (Page 2, line 41-45)
4. However, there is still limited understanding of the complex nature of father-child relationships, especially when considering cultural, socioeconomical and family structures. (Page 3, line 53-55)
5. This scoping review will report the characteristics of father-child relationship, across the entire range of childhood and amongst neurotypical and neurodiverse children. (Page 13, line 284-285)
6. The knowledge-base, synthesised by this review, will inform the development of a framework for paternal involvement that is applicable across majority family contexts. (Page 19, line 368-369)

7. Is a review incorporating only qualitative and mixed methods projects really suitable to answer the question: "What are the socio-demographic determinants of paternal involvement?" Response: Thank you for your thoughtful comment. Existing research has extensively examined the quantitative associations between paternal involvement and child developmental, and socio-economic outcomes (McWayne et al., 2013; Rollè et al., 2019; Sarkadi A et al., 2008). However, there is limited synthesis of the qualitative literature in this area. As scoping reviews are well suited to synthesising qualitative evidence across conceptually heterogeneous studies, the key gap lies in the integration of qualitative findings rather than further quantitative analysis (Arksey & O’Malley, 2005; Levac et al., 2010; M. D. J. Peters et al., 2015, 2020) We would like to clarify that the term *“determinants”* is not used here in the conventional quantitative sense, where it typically refers to effect sizes or measured associations (e.g., socio-economic status and levels of father involvement). Instead, in this review, we use the term to describe how socio-demographic factors are experienced, interpreted, and understood within father–child relationships We recognise the need for clarity, and in the revised protocol manuscript made the changes as follows: In the next step reviewers will examine these studies to explore how socio-demographic factors are described, experienced and understood within father-child relationships, as well as how paternal involvement differs across high vs low-resource settings, early vs late childhood and among neurotypical vs neurodiverse children, in order to address the secondary research questions of this review. (Page 18, line 347-351)

 8. If a study is considered to have a high risk of bias from CASP, will it be included in the scoping review or no? Some explanation of this is needed. Response:  Yes, it will be included. We added an explanation for 8 in the manuscript as follows: All eligible studies will be included in the review, including those assessed as having a high risk of bias using the CASP tool, as their findings may still offer valuable insights into the characteristics, contexts, and conceptualisations of father involvement within specific geographical and cultural settings. To ensure transparency and support appropriate interpretation of the findings, risk-of-bias assessments will be reported alongside each study, and any limitations identified through CASP will be clearly highlighted. (Page 18, line 333-338)  

 Small points:

 - Appears to be a typo here: "This scoping review involves five stages" Response: Thank you for pointing out. We changed it in the revised draft – This scoping review involves five stages. (page 2, line 23).  

 - I think "The" or "An" is missing here: "Electronic database search will be performed on PubMed and PsycINFO..." Response: Thank you for pointing out. We changed it in the revised draft - An electronic database search will be performed on PubMed and PsycINFO…(page 2, line 24-25).

 - Research from more than 10 years ago is not really "emerging literature": "Emerging literature demonstrates that positive child social, emotional, psychosocial and developmental outcomes are associated with high-quality paternal involvement ( Gibson, 2014)." also, there is a long-standing and rapidly growing body of work on this topic which this sentence ignores.  Response: Thank you for pointing out. We added the recent references in the revised draft - Emerging literature demonstrates that positive child social, emotional, psychosocial and developmental outcomes are associated with high-quality paternal involvement (Abraham E et al., 2021; Diniz et al., 2021; Gibson, 2014; Kenigsberg et al., 2016; Solichah et al., 2025). (page 4, line 87-88).

 - "the conceptualisation of paternal involvement evolved as Hawkins’ *et al.* ( Hawkins *et al.*, 2002)" not correct formatting for the reference. Similar issue here: "Lut *et al.* ( Lut *et al.*, 2022)" and "Diniz E *et al.* ( Diniz *et al.*, 2021)" etc Response: Thank you for pointing out. We formatted the correct references in the revised draft – the conceptualisation of paternal involvement evolved as Hawkins’ et al., 2002 (page 5, line – 102) turning in refers to father’s emotional connect with their child for understanding and responding to their needs and lastly mentorship refers to father’s role as mentor and guide for the child (Hawkins et al., 2002). (page 5, line – 109) Lut et al., 2022 conducted a systematic scoping review of studies of fatherhood that used linked administrative data to their children and studies outcomes were related to child health and development (Lut et al., 2022). (page 6, line – 125, 127) Diniz E et al., 2021 conducted a systematic review of qualitative studies examining father involvement during early childhood. This review highlighted the significance of paternal engagement in shaping child development outcomes, revealing themes such as emotional support, playfulness, and co-parenting dynamics (Diniz et al., 2021).  (page 6, line - 130, 134)

 - Typo here: "such as paternal engagement or attachment and that too during early-childhood" Response: Thank you for pointing out. We corrected the mentioned typo in the revised draft – such as paternal engagement or attachment and that too during early childhood (page 7, line 144-145).

 - Typo here: "Participants demographics" Response: Thank you for pointing out. We corrected the mentioned typo in the revised draft – Participants demographic (page 11, line 243). Once again, thank you for these valuable suggestions.

**Updated references **

1. Arksey, H., & O’Malley, L. (2005). Scoping studies: Towards a methodological framework. *International Journal of Social Research Methodology*, *8*(1), 19–32. https://doi.org/10.1080/1364557032000119616
2. Berkel C, Sandler IN, Wolchik SA, Brown CH, Gallo CG, Chiapa A, Mauricio AM, & Jones S. (2018). “Home Practice Is the Program”: Parents’ Practice of Program Skills as Predictors of Outcomes in the New Beginnings Program Effectiveness Trial. *Prevention Science : The Official Journal of the Society for Prevention Research*, *19*(5), 663–673. https://doi.org/10.1007/s11121-016-0738-0
3. Bronstein, P. (2006). The Family Environment: Where Gender Role Socialization Begins. *Handbook of Girls’ and Women’s Psychological Health: Gender and Well-Being across the Lifespan.*, 262–271.
4. Bronte-Tinkew, J., Scott, M. E., Horowitz, A., & Lilja, E. (2009). Pregnancy intentions during the transition to parenthood and links to coparenting for first-time fathers of infants. *Parenting: Science and Practice*, *9*(1), 1–35. https://doi.org/10.1080/15295190802656729
5. *Guidelines on Mental Health Promotive and Preventive Interventions for Adolescents: Helping Adolescents Thrive* (1st ed). (2020). World Health Organization.
6. Lamb, M. E. (n.d.-a). *The Role of the Father in Child Development, Fifth Edition*.
7. Lamb, M. E. (n.d.-b). *The Role of the Father in Child Development, Fifth Edition*.
8. Levac, D., Colquhoun, H., & O’Brien, K. K. (2010). Scoping studies: Advancing the methodology. *Implementation Science*, *5*(1), 69. https://doi.org/10.1186/1748-5908-5-69
9. McWayne, C., Downer, J. T., Campos, R., & Harris, R. D. (2013). Father involvement during early childhood and its association with children’s early learning: A meta-analysis. *Early Education and Development*, *24*(6), 898–922. https://doi.org/10.1080/10409289.2013.746932
10. Moher, D., Stewart, L., & Shekelle, P. (2015). All in the Family: Systematic reviews, rapid reviews, scoping reviews, realist reviews, and more. *Systematic Reviews*, *4*(1), 183, s13643-015-0163–0167. https://doi.org/10.1186/s13643-015-0163-7
11. Moore, T. (2003). Handbook of Father Involvement: Multidisciplinary Perspectives: *Journal of Developmental & Behavioral Pediatrics*, *24*(4), 292–293. https://doi.org/10.1097/00004703-200308000-00013
12. Munn, Z., Peters, M. D. J., Stern, C., Tufanaru, C., McArthur, A., & Aromataris, E. (2018). Systematic review or scoping review? Guidance for authors when choosing between a systematic or scoping review approach. *BMC Medical Research Methodology*, *18*(1), 143. https://doi.org/10.1186/s12874-018-0611-x
13. Peters, M. D. J., Godfrey, C. M., Khalil, H., McInerney, P., Parker, D., & Soares, C. B. (2015). Guidance for conducting systematic scoping reviews. *International Journal of Evidence-Based Healthcare*, *13*(3), 141–146. https://doi.org/10.1097/XEB.0000000000000050
14. Peters, M. D. J., Marnie, C., Tricco, A. C., Pollock, D., Munn, Z., Alexander, L., McInerney, P., Godfrey, C. M., & Khalil, H. (2020). Updated methodological guidance for the conduct of scoping reviews. *JBI Evidence Synthesis*, *18*(10), 2119–2126. https://doi.org/10.11124/JBIES-20-00167
15. Peters, S. S. (2021). *Relations among interparental relationship quality, maternal depressive symptoms, parenting in the home environment, and pre-school child behavior in African American, low-income, single-mother families.* (rayyan-1253282433). *82*(11), No Pagination Specified-No Pagination Specified.
16. Rollè, L., Gullotta, G., Trombetta, T., Curti, L., Gerino, E., Brustia, P., & Caldarera, A. M. (2019). Father Involvement and Cognitive Development in Early and Middle Childhood: A Systematic Review. *Frontiers in Psychology*, *10*, 2405. https://doi.org/10.3389/fpsyg.2019.02405
17. Santrock, J. W. (2019). *Children* (Fourteenth edition). McGraw-Hill Education.
18. Sarkadi A, Kristiansson R, Oberklaid F, & Bremberg S. (2008). Fathers’ involvement and children’s developmental outcomes: A systematic review of longitudinal studies. *Acta Paediatrica (Oslo, Norway : 1992)*, *97*(2), 153–158. https://doi.org/10.1111/j.1651-2227.2007.00572.x
19. Tricco, A. C., Lillie, E., Zarin, W., O’Brien, K. K., Colquhoun, H., Levac, D., Moher, D., Peters, M. D. J., Horsley, T., Weeks, L., Hempel, S., Akl, E. A., Chang, C., McGowan, J., Stewart, L., Hartling, L., Aldcroft, A., Wilson, M. G., Garritty, C., … Straus, S. E. (2018). PRISMA Extension for Scoping Reviews (PRISMA-ScR): Checklist and Explanation. *Annals of Internal Medicine*, *169*(7), 467–473. https://doi.org/10.7326/M18-0850

# Reviewer report: Reviewer response for version 1

**DOI:** 10.21956/wellcomeopenres.26062.r130072

**Published:** 2025-09-08

**Authors:** Lucia Lombardi

**Affiliations:** 1 University "G. d'Annunzio" of Chieti-Pescara, Pescara, Italy

This work shows a detailed and well-structured protocol for a scoping review on the characteristics of father-child relationships across diverse cultural, socioeconomic, and family contexts. The protocol addresses an important and timely research area, given the increasing recognition of fathers’ roles in child development and mental health.

 In my opinion the review is well written and well structured, maybe the wide range of settings, child age groups, and inclusion of neurodiverse populations may pose challenges in achieving depth of analysis. I think that Authors shiuld include this point in a limitations section.

Is the study design appropriate for the research question?

Yes

Is the rationale for, and objectives of, the study clearly described?

Yes

Are sufficient details of the methods provided to allow replication by others?

Yes

Are the datasets clearly presented in a useable and accessible format?

Yes

Reviewer Expertise:

Clinical Psychology, parenthood, genetic predisposition

I confirm that I have read this submission and believe that I have an appropriate level of expertise to confirm that it is of an acceptable scientific standard.

# Response

**Published:** 2026-01-11

**Authors:** SMITA TODKAR

**Affiliations:** Child Development Group (CDG), Sangath, Porvorim, Goa, India

Thank you for your review.  We will note as a limitation the restricted depth of analysis due to the wide range of settings, child age group and inclusion of neurodiverse populations.
